# Magnetic interactions between nanoparticles

**DOI:** 10.3762/bjnano.1.22

**Published:** 2010-12-28

**Authors:** Steen Mørup, Mikkel Fougt Hansen, Cathrine Frandsen

**Affiliations:** 1 Department of Physics, Building 307; Technical University of Denmark; DK-2800 Kongens Lyngby; Denmark; 2 Department of Micro- and Nanotechnology, DTU Nanotech, Building 345 East; Technical University of Denmark; DK-2800 Kongens Lyngby; Denmark

**Keywords:** dipole interactions, exchange interactions, spin structure, superferromagnetism, superparamagnetic relaxation

## Abstract

We present a short overview of the influence of inter-particle interactions on the properties of magnetic nanoparticles. Strong magnetic dipole interactions between ferromagnetic or ferrimagnetic particles, that would be superparamagnetic if isolated, can result in a collective state of nanoparticles. This collective state has many similarities to spin-glasses. In samples of aggregated magnetic nanoparticles, exchange interactions are often important and this can also lead to a strong suppression of superparamagnetic relaxation. The temperature dependence of the order parameter in samples of strongly interacting hematite nanoparticles or goethite grains is well described by a simple mean field model. Exchange interactions between nanoparticles with different orientations of the easy axes can also result in a rotation of the sub-lattice magnetization directions.

## Review

### Introduction

In nanostructured magnetic materials, interactions between, for example, nanoparticles or thin films in multilayer structures often play an important role. Long-range magnetic dipole interactions can have a strong influence on, e.g., the magnetic dynamics in samples containing ferromagnetic or ferrimagnetic nanoparticles. If nanoparticles or thin films are in close proximity, exchange interactions between surface atoms can be significant. An important example of magnetic proximity effects is exchange bias, which manifests itself as a shift of the hysteresis curves obtained after field cooling of a ferromagnetic or ferrimagnetic material in contact with an antiferromagnetic material [1–3]. This was first observed in nanoparticles consisting of a core of ferromagnetic cobalt covered by a shell of antiferromagnetic CoO [4]: This effect is nowadays utilized in read heads in computer hard disk drives. In a neutron study of Fe_3_O_4_/CoO multilayers, van der Zaag et al. [5] found that the Néel temperature of CoO was enhanced due to the exchange interaction with ferrimagnetic Fe_3_O_4_ layers with a Curie temperature of about 850 K. Similarly, an increase of the Curie temperature of ferrimagnetic γ-Mn_2_O_3_ due to interaction with antiferromagnetic MnO has been found in MnO/γ-Mn_2_O_3_ core–shell particles [6].

The magnetic properties of non-interacting magnetic nanoparticles are often strongly influenced by superparamagnetic relaxation at finite temperatures. For a nanoparticle with uniaxial anisotropy and with the magnetic anisotropy energy given by the simple expression

[1]
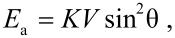


there are energy minima at θ = 0° and θ = 180°, which are separated by an energy barrier *KV*. Here *K* is the magnetic anisotropy constant, *V* is the particle volume and θ is the angle between the magnetization vector and an easy direction of magnetization. At finite temperatures, the thermal energy may be sufficient to induce superparamagnetic relaxation, i.e., reversal of the magnetization between directions close to θ = 0° and θ = 180°. The superparamagnetic relaxation time is given by the Néel–Brown expression [7–8]

[2]
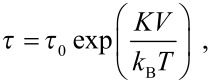


where *k*_B_ is Boltzmann’s constant and *T* is the temperature. τ_0_ is on the order of 10^−13^–10^−9^ s and is weakly temperature dependent.

In experimental studies of magnetic nanoparticles, the timescale of the experimental technique is an important parameter. If the relaxation is fast compared to the timescale of the experimental technique one measures an average value of the magnetization, but if the relaxation time is long compared to the timescale of the experimental technique, one measures the instantaneous value of the magnetization. The superparamagnetic blocking temperature is defined as the temperature at which the superparamagnetic relaxation time equals the timescale of the experimental technique. In Mössbauer spectroscopy the timescale is on the order of a few nanoseconds, whereas it is on the order of picoseconds in inelastic neutron scattering studies. In DC magnetization measurements the timescale is in the range 1–100 seconds. In AC magnetization measurements the timescale can be varied by varying the frequency. Thus, the blocking temperature is not uniquely defined, but it depends on the timescale of the experimental technique.

If magnetic interactions between the particles are not negligible, they can have a significant influence on the superparamagnetic relaxation. Furthermore, the spin structure of nanoparticles can be affected by inter-particle interactions. In this short review, we first discuss how the superparamagnetic relaxation in nanoparticles can be influenced by magnetic dipole interactions and by exchange interactions between particles. Subsequently, we discuss how the spin structure of nanoparticles can be influenced by inter-particle exchange interactions.

### Magnetic dipole interactions

Magnetic dipole interactions between atoms in crystals with magnetic moments of a few Bohr magnetons are too small to result in magnetic ordering above 1 K and are usually negligible compared to exchange interactions in magnetic materials. Therefore, magnetic dipole interactions have a negligible influence on the magnetic order in bulk materials at finite temperatures. However, nanoparticles of ferromagnetic and ferrimagnetic materials with dimensions around 10 nm can have magnetic moments larger than 10,000 Bohr magnetons, and therefore, dipole interactions between nanoparticles can have a significant influence on the magnetic properties.

In a sample of randomly distributed nanoparticles with average magnetic moment μ and average separation *d*, the dipole interaction energy of a particle is on the order of [9]

[3]
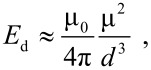


where μ_0_ is the permeability of free space. In samples with high concentrations of magnetic nanoparticles, which would be superparamagnetic if they were non-interacting, magnetic dipole interactions can result in ordering of the magnetic moments of the nanoparticles below a critical temperature *T*_0_, where [9]

[4]
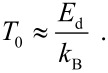


Systems of magnetic nanoparticles with only magnetic dipole interactions can be prepared by dispersing magnetic nanoparticles coated with surfactant molecules in a solvent. Often, nanoparticles have a broad size distribution that gives rise to a very broad distribution of superparamagnetic relaxation times of the isolated particles (Equation 2). To distinguish effects of single particle behavior from those of inter-particle interactions, a very narrow particle size distribution is required. Interparticle interactions can be varied by changing the concentration of the particles and can be studied in frozen samples. A wide variety of nanoparticle systems, including Fe_100−_*_x_*C*_x_* [10], ε-Fe_3_N [11], γ-Fe_2_O_3_ [12–14] and Fe_3_O_4_ [15] have been investigated. If the particles are randomly distributed and have a random orientation of the easy axes, the magnetic properties can have similarities to those of spin glasses [10–11,14], and therefore these interacting nanoparticle systems are often called super-spin glasses.

Dipole interactions can have a significant influence on DC magnetization measurements. In zero field cooled (ZFC) magnetization studies one measures the temperature dependence of the magnetization in a small applied field after the sample has been cooled in zero field. Samples of non-interacting particles show a maximum in the ZFC curve at a temperature *T*_p_ related to the blocking temperature. Dipole interactions result in a shift of the maximum to a higher temperature. Field cooled (FC) magnetization curves are obtained in a similar way, but after cooling the sample in a small field. For samples of non-interacting particles, the FC magnetization curve increases with decreasing temperature below *T*_p_, but interactions can result in an almost temperature independent magnetization below *T*_p_. Such measurements have been used to investigate interaction effects in numerous studies, e.g., [11–13], and are useful for a qualitative characterization of samples of interacting nanoparticles. However, it is difficult to obtain quantitative information on the influence of interactions from DC magnetization measurements.

AC magnetization measurements can be used to obtain quantitative information on the relaxation time. Such measurements on samples of interacting nanoparticles have shown that the relaxation time diverges in the same manner as in a spin glass, when the sample is cooled towards the phase transition temperature *T*_0_ [10,14,16–18], i.e., the relaxation time can be expressed by

[5]
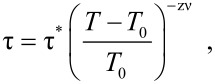


where τ* is the relaxation time of non-interacting particles and the critical exponent zν is on the order of 10. Another sign of spin-glass-like behavior is a divergence of the non-linear magnetic susceptibility when *T*_0_ is approached from above [11,19]. Moreover, below *T*_0_ the memory and rejuvenation phenomena that are characteristic for spin-glass behavior have been observed [20]. The studies of ‘super spin-glass’ behavior have recently been reviewed [21–22].

**Figure 1:**
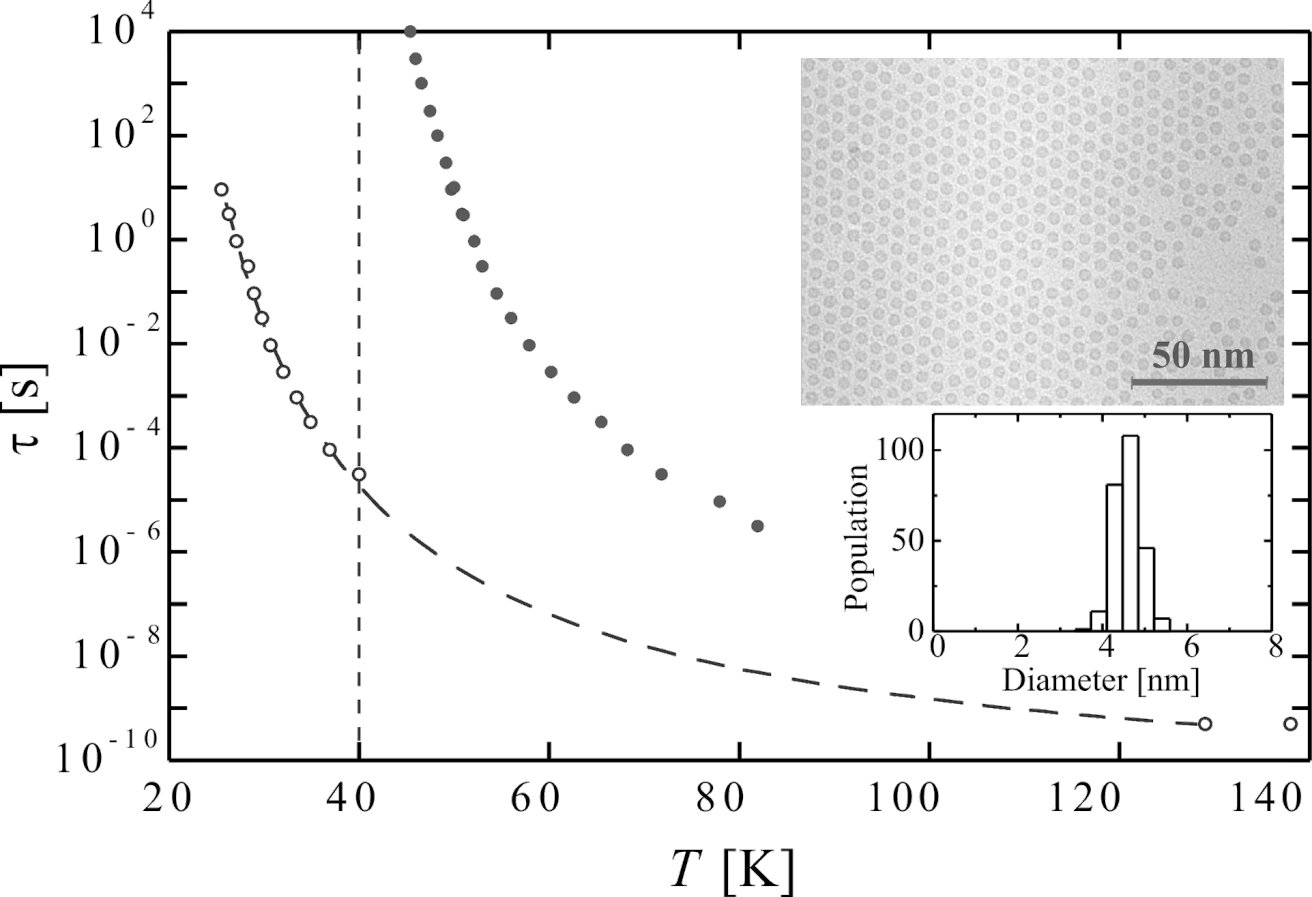
The relaxation time of 4.7 nm Fe_100−_*_x_*C*_x_* nearly monodisperse particles suspended in decalin as a function of temperature. The data were obtained from AC susceptibility measurements. The open circles are data from a dilute sample, whereas the full circles are data for a concentrated sample. The insets show a transmission electron microscopy (TEM) image of the particles deposited on an amorphous carbon film and the corresponding particle size distribution obtained from the TEM images. Adapted from Djurberg, C.; Svedlindh, P.; Nordblad, P.; Hansen, M. F.; Bødker, F.; Mørup, S. Dynamics of an Interacting Particle System: Evidence of Critical Slowing Down, *Phys. Rev. Lett.* **1997,*** 79,* 5154. Copyright (1997) by the American Physical Society.

As an example, Figure 1 shows the relaxation time of suspensions of nearly monodisperse 4.7 nm Fe_100−_*_x_*C*_x_* particles (*x* ≈ 22) in decalin as a function of temperature. The data were obtained from AC susceptibility measurements. The open circles are data from a dilute sample, whereas the full circles are data for a concentrated sample. The temperature dependence of the relaxation time for the dilute sample is in accordance with Equation 2, whereas the temperature dependence of the relaxation time of the concentrated sample is in accordance with Equation 5, and the relaxation time diverges at *T*_0_ = 40 K [10]. The insets show an electron micrograph of the particles and the particle size distribution.

Granular systems with a different content of metallic nanoparticles, e.g., Co [23] or Co_80_Fe_20_ [24] embedded in a non-magnetic matrix, have been prepared by sputtering of discontinuous metal–insulator multi-layers and subsequent annealing. These systems have shown both spin-glass-like ordering for moderately strong interactions and ferromagnetic ordering for very strong interactions [24]. The latter transition is attributed to a weak exchange coupling through magnetic impurities in the insulating matrix [24]. Similarly, in the Fe*_x_*Ag_100−_*_x_* granular system of 2.5–3.0 nm Fe particles in an Ag matrix, a cross-over was observed from a spin-glass-like behavior of the particle moments for *x* < 35 to a ferromagnetic ordering of the particle moments for 35 < *x* < 50 [25]. In this system, the magnetic particles also interact via the RKKY interaction because of the conducting Ag matrix.

Often, there is a tendency for magnetic nanoparticles to form chains, especially if they can move freely in an external magnetic field, for example, if they are suspended in a liquid. If the nanoparticles form chains, a ferromagnetic ordering of the magnetic moments is favored in zero applied field with the magnetization along the chain direction [26–27]. Using a mean field model for an infinite chain of interacting nanoparticles with separation *d*, one finds that the ordering temperature is given by [27]

[6]
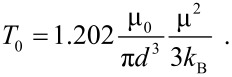


Thus, in general, strong dipole interactions result in suppression of the superparamagnetic relaxation. It is, however, remarkable that weak dipole interactions can result in faster superparamagnetic relaxation. This has been observed in Mössbauer studies of maghemite (γ-Fe_2_O_3_) nanoparticles [12,28], and the effect has been explained by a lowering of the energy barriers between the two minima of the magnetic energy [28–31].

**Figure 2:**
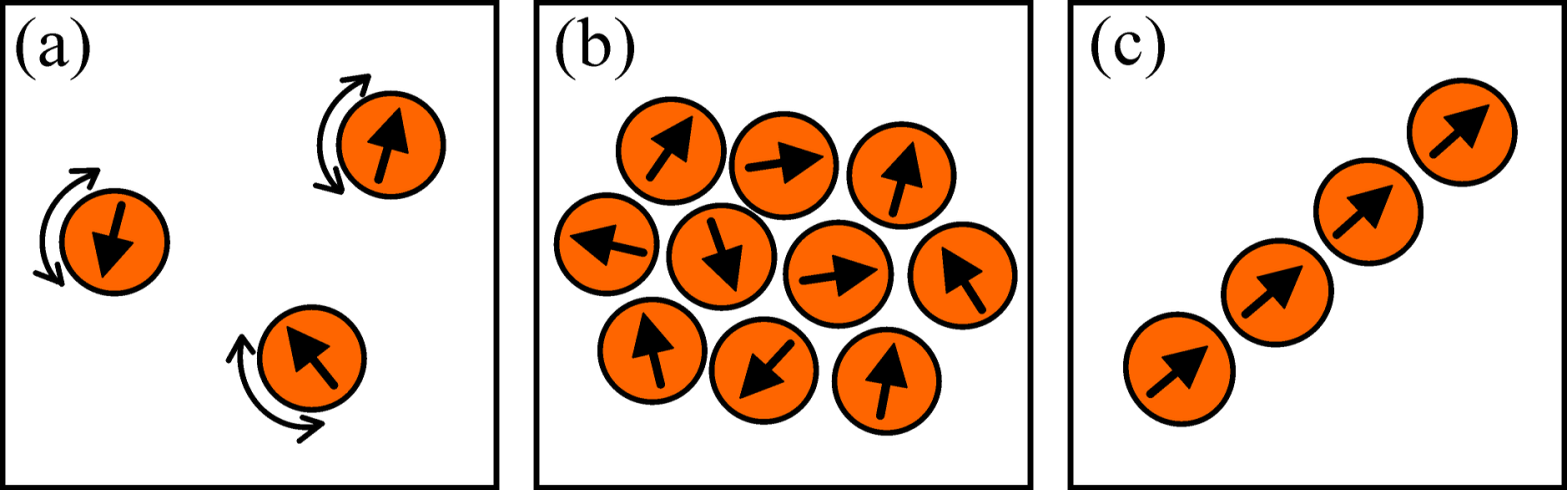
Schematic illustration of interacting magnetic nanoparticles. (a) Isolated nanoparticles dominated by superparamagnetic relaxation. (b) Interacting nanoparticles forming a dipole glass. (c) Nanoparticles forming a chain with aligned dipole moments.

Figure 2 shows a schematic illustration of interacting nanoparticles. Figure 2a illustrates isolated nanoparticles, dominated by superparamagnetic relaxation. Figure 2b shows interacting nanoparticles forming a “dipole glass”. The nanoparticles in Figure 2c form a chain with aligned dipole moments.

By the use of off-axis electron holography, it is possible to obtain information about the magnetization direction of individual nanoparticles in ensembles of interacting ferro- or ferrimagnetic nanoparticles. This technique measures quantitatively and non-invasively the in-plane magnetic field component of a thin sample with a lateral resolution of a few nanometers [32–33]. From the obtained images, the influence of dipolar interactions between magnetic nanoparticles can be very apparent. For example, this technique has resolved an almost linear magnetic flux along the chain direction in a double chain of 24 ~70 nm magnetite (Fe_3_O_4_) particles in magnetotactic bacteria [33], and it has resolved magnetic flux closure in small rings of 5–7 Co particles with a diameter of about 25 nm [32].

### Influence of exchange coupling between nanoparticles on magnetic relaxation

**Figure 3:**
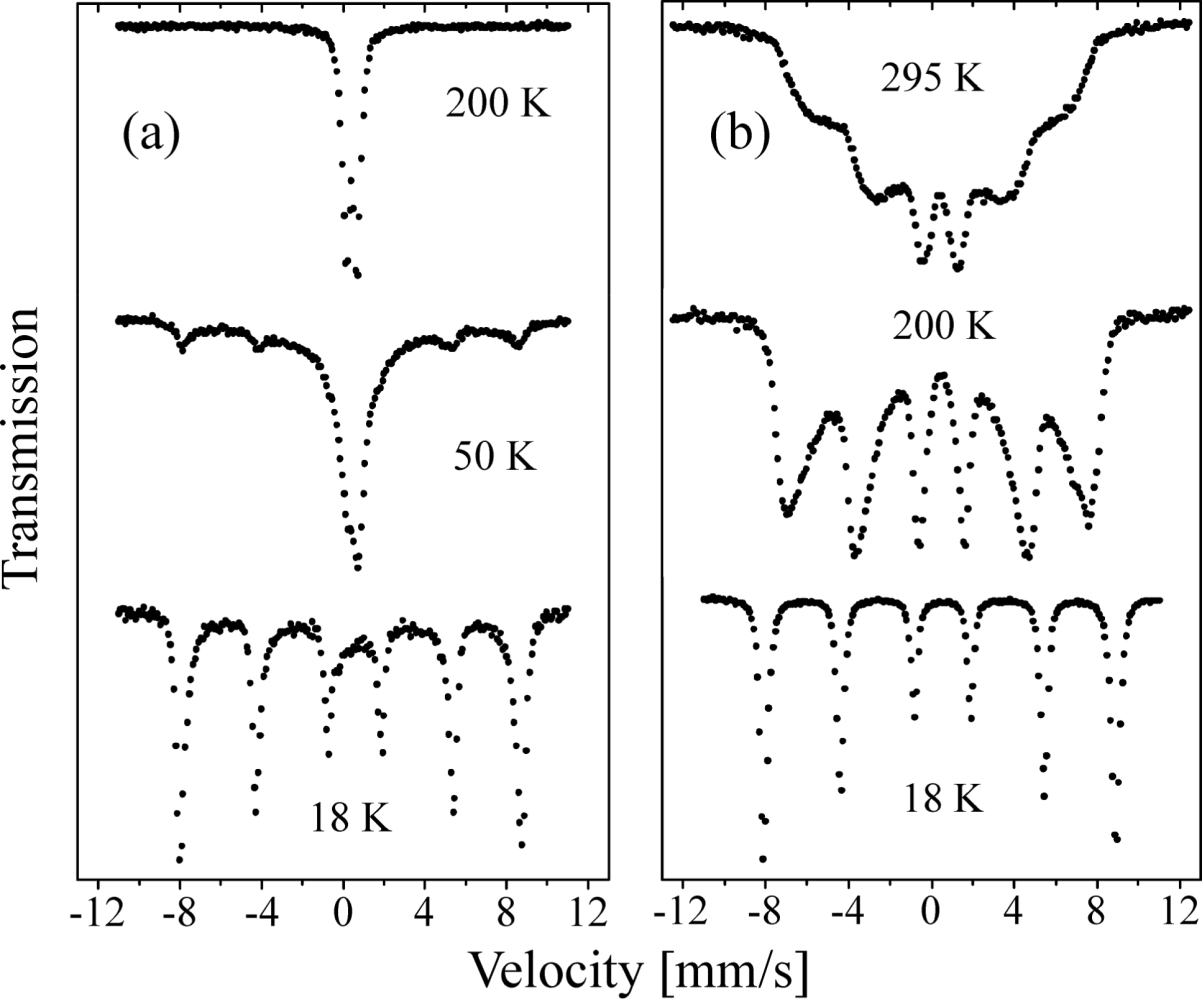
Mössbauer spectra of 8 nm hematite particles (a) coated (non-interacting) and (b) uncoated (strongly interacting) nanoparticles. The spectra were obtained at the indicated temperatures. Reprinted from Frandsen, C.; Mørup, S. Spin rotation in α-Fe_2_O_3_ nanoparticles by interparticle interactions, *Phys. Rev. Lett*. **2005,** *94,* 027202. Copyright (2005) by the American Physical Society.

In a perfect antiferromagnetic material the net magnetization vanishes because the sublattice magnetizations have identical size but opposite directions. However, in nanoparticles, the finite number of magnetic ions results in a small net magnetic moment because of uncompensated spins in the surface and/or in the interior of the particles [34]. This magnetic moment is, however, usually so small that dipole interactions are almost negligible and the influence of dipole interactions on the superparamagnetic relaxation is therefore also expected to be negligible [35]. Nevertheless, several Mössbauer studies of, for example, hematite (α-Fe_2_O_3_) [35–38] and ferrihydrite [39] nanoparticles have shown that the superparamagnetic relaxation of antiferromagnetic nanoparticles can be significantly suppressed if the particles are in close proximity. This has been explained by exchange interaction between surface atoms of neighboring particles [35–38]. As an example, Figure 3 shows Mössbauer spectra of chemically prepared 8 nm hematite (α-Fe_2_O_3_) nanoparticles [36]. The spectra in Figure 3a were obtained from particles, which were coated with phosphate in order to minimize inter-particle interactions. The spectra in Figure 3b were obtained from a sample prepared by freeze-drying an aqueous suspension of uncoated particles from the same batch. At 18 K, the spectra of both coated and uncoated particles consist of a sextet with relatively narrow lines, indicating that relaxation effects are negligible. At 50 K the spectrum of the coated particles in Figure 3a show a superposition of a sextet and a doublet, which are due to particles below and above their blocking temperature, respectively. Both the sextet and the doublet have relatively narrow lines. The relative area of the doublet increases with increasing temperature at the expense of the sextet. At 200 K the sextet has disappeared and the spectra only show a quadrupole doublet, indicating that all particles show fast superparamagnetic relaxation (τ < 1 ns). The presence of both a sextet and a doublet in the spectra in Figure 3a and the temperature dependence of the relative areas can be explained by the particle size distribution in combination with the exponential dependence of the relaxation time on the particle volume (Equation 2). In Mössbauer spectroscopy studies of magnetic nanoparticles the median blocking temperature of a sample is usually defined as the temperature where half of the spectral area is in the sextet and the remaining area is in the doublet.

The spectra of the dried, uncoated particles in Figure 3b show a quite different temperature dependence. As the temperature is increased, the lines gradually broaden and the average hyperfine field decreases, but even at 295 K there is no visible doublet in the spectrum. This shows that the superparamagnetic relaxation is strongly suppressed compared to the sample of coated particles. Thus, the different evolution of the spectra as a function of temperature clearly shows that the magnetic relaxation is qualitatively different in samples of non-interacting and interacting nanoparticles.

In several earlier publications it was assumed that the magnetic interactions between nanoparticles can be treated as an extra contribution to the magnetic anisotropy. If this were correct, the Mössbauer spectra of non-interacting and interacting particles should be qualitatively similar and the only difference should be a higher median superparamagnetic blocking temperature in samples of interacting nanoparticles. The different temperature dependence of the spectra in Figure 3a and Figure 3b shows that this assumption is incorrect. As discussed below, the influence of inter-particle interactions should rather be treated in terms of an interaction field [35,37,40].

Mössbauer data for strongly interacting antiferromagnetic particles have been analyzed using a “superferromagnetism” model [35,40], in which it is assumed that the magnetic energy of a particle, interacting with its neighbors, is given by

[7]
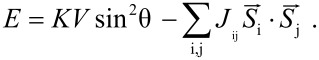


Here, the first term represents the magnetic anisotropy energy. The second term is the interaction energy, where 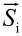 and 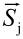 are the surface spins belonging to the particle and the neighboring particles, respectively, and *J*_ij_ is the exchange coupling constant. The summation in Equation 7 may be replaced by a mean field, acting on the sublattice magnetization of the particle [35,37,40]

[8]
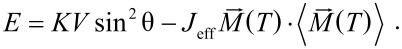



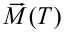
 represents the sub-lattice magnetization vector of a particle at temperature *T*, *J_eff_* is an effective exchange coupling constant and 
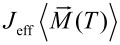
 is an effective interaction mean field acting on 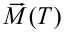.

**Figure 4:**
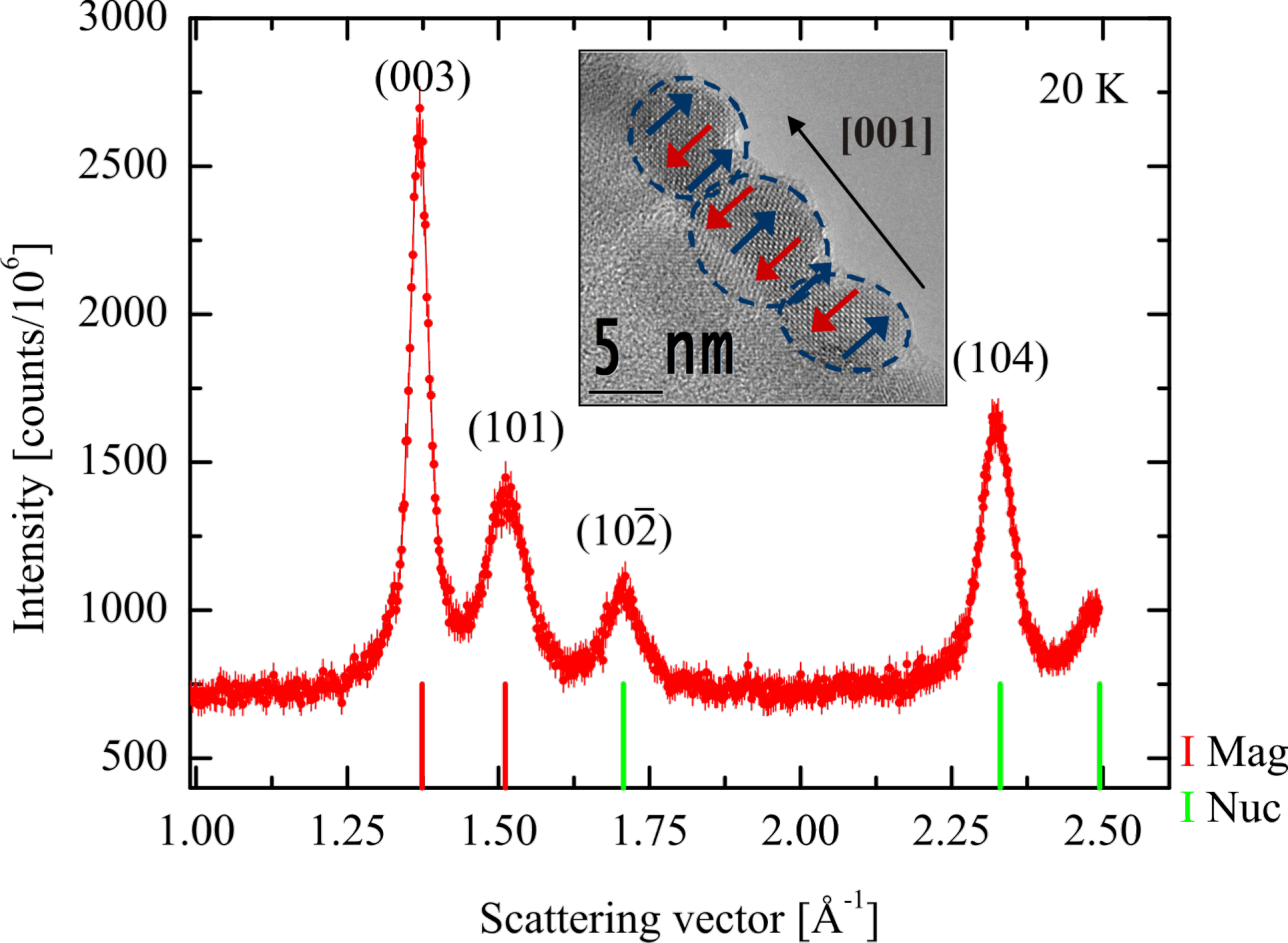
Neutron diffraction data for interacting 8 nm α-Fe_2_O_3_ particles obtained at 20 K. The inset shows a TEM image of three α-Fe_2_O_3_ particles attached along their common [001] axis. The antiferromagnetic order is indicated by the blue and red arrows superimposed on the TEM image. Adapted from Frandsen, C.; Bahl, C. R. H.; Lebech, B.; Lefmann, K.; Kuhn, L. T.; Keller, L.; Andersen, N. H.; von Zimmermann, M.; Johnson, E.; Klausen, S. N.; Mørup, S. Oriented attachment and exchange coupling of α-Fe_2_O_3_ nanoparticles, *Phys. Rev. B* **2005,*** 72,* 214406. Copyright (2005) by the American Physical Society.

The magnetic energy (Equation 8) will depend on the angle between the easy axis, defined by the magnetic anisotropy and the interaction field. In recent studies it has been found that chemically prepared nanoparticles of antiferromagnetic hematite can in some cases be attached with a common orientation such that both the crystallographic and the magnetic order continue across the interface [38]. This is illustrated by the neutron diffraction data for 8 nm hematite nanoparticles prepared by freeze drying an aqueous suspension of uncoated particles, shown in Figure 4 [41]. The particles were prepared chemically by means of a method similar to the D-preparation described by Sugimoto et al. [42]. As in X-ray diffraction studies, the peaks in the neutron diffraction patterns of these nanoparticles are broadened, and the broadening is related to the crystallographic and the magnetic correlation lengths as described by the Scherrer formula [38]. The width of most of the neutron diffraction lines in Figure 4 is in accordance with the particle size estimated from electron microscopy. However, the purely magnetic (003) peak is considerably narrower than the other peaks [38,41]. This shows that the magnetic correlation length in this direction is larger than the particle size, i.e., the magnetic (and the crystallographic) correlation extends over several particles. After gentle grinding, neutron diffraction studies showed that the width of the (003) peak becomes similar to those of the other peaks, indicating that the oriented attachment is destroyed [38]. In studies of nanoparticles of goethite (α-FeOOH) [40,43–44] it has also been found that there is a tendency for (imperfect) oriented attachment of grains.

When particles are attached with a common orientation, it may be a good first order approximation to assume that the interaction field and the anisotropy field are parallel [35,40] such that Equation 8 can be replaced by

[9]
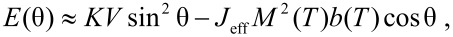


where *M(T)* is the sub-lattice magnetization in the absence of magnetic fluctuations, and

[10]
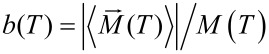


is the order parameter.

**Figure 5:**
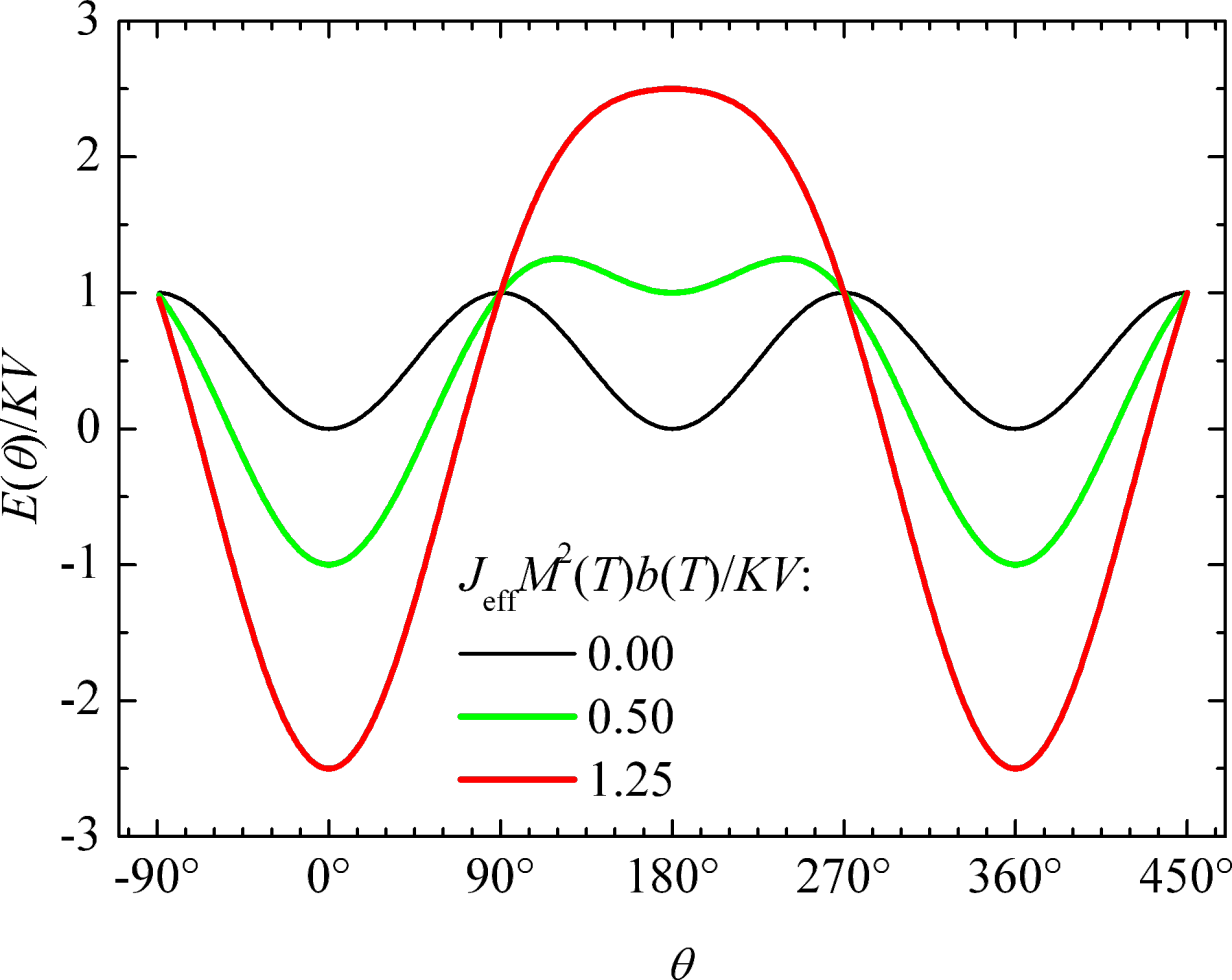
The normalized magnetic energy, *E*(θ)/*KV* (Equation 9) for different values of the ratio between the interaction energy *J*_eff_*M*^2^(*T*)*b*(*T*) and the anisotropy energy, *KV*.

The magnetic energy, *E*(θ) (Equation 9) is shown in Figure 5 for different values of the ratio between the interaction energy *J*_eff_*M*^2^(*T*)*b*(*T*) and the anisotropy energy, *KV*. If the interaction energy is negligible compared to the anisotropy energy, the relaxation can be described in terms of transitions between the minima at 0° and 180°, but if the interaction energy is predominant, there is only one minimum, defined by the effective interaction field and the anisotropy. In the presence of a finite interaction field, there may be two minima with different energies. Then the average value of the sublattice magnetization is non-zero, and therefore a magnetic splitting appears in Mössbauer spectra even at high temperatures where the relaxation is fast. In thermal equilibrium, i.e., when all relaxation processes can be considered fast compared to the timescale of the Mössbauer spectroscopy, the temperature dependence of the order parameter can be calculated by use of Boltzmann statistics [35,40]

[11]
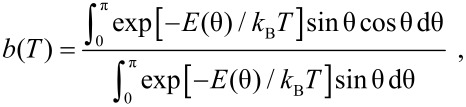


where *E*(θ) is given by Equation 9. Equation 11 can be solved numerically to estimate the temperature dependence of the order parameter. If the relaxation is fast compared to the timescale of Mössbauer spectroscopy, the magnetic hyperfine splitting in the spectra will be proportional to *b*(*T*). In samples where the magnetic anisotropy energy can be considered negligible compared to the interaction energy, the magnetic ordering of the particle moments will disappear at the ordering temperature given by

[12]
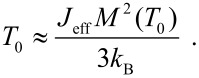


The superferromagnetism model has been successfully used to fit data for interacting nanoparticles of hematite [35] and goethite grains [40].

**Figure 6:**
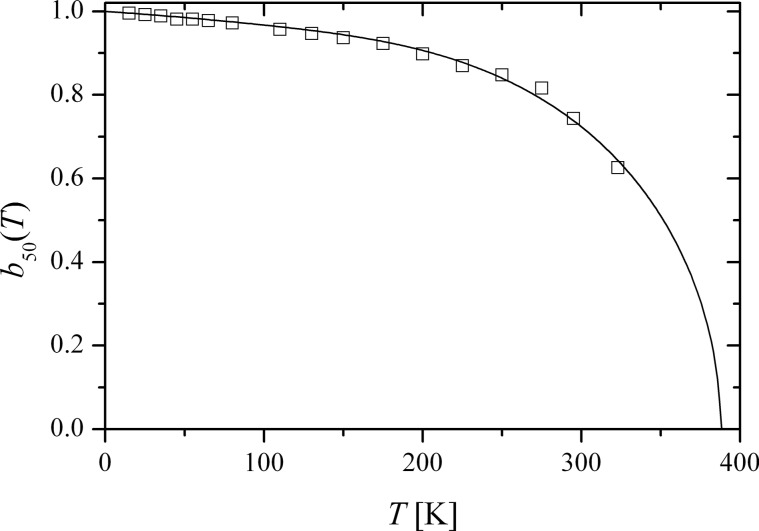
Temperature dependence of the median value of the order parameter, *b*_50_(*T*) for interacting 20 nm hematite nanoparticles. The open squares are the experimental data, and the solid line is a fit to the superferromagnetism model (Equation 11). Adapted from Hansen, M. F. ; Koch, C. B.; Mørup, S. Magnetic dynamics of weakly and strongly interacting hematite nanoparticles, *Phys. Rev. B* **2000,** *62,* 1124. Copyright (2000) by the American Physical Society.

The variation in the local environments of the particles in a sample results in a distribution of the magnitudes of the order parameters. Consequently, the value of the order parameter at a given temperature is not the same for all parts of the sample, and this leads to a distribution of magnetic hyperfine fields, which explains the line broadening in the spectra. It is convenient to analyze the temperature dependence of chosen quantiles of the hyperfine field distribution when comparing with the theoretical superferromagnetism model (Equation 11) [35,40]. Figure 6 shows the temperature dependence of the order parameter, *b*_50_(*T*) of the 50% quantile of the hyperfine field distribution (the median hyperfine field) for interacting 20 nm hematite nanoparticles. The solid line is a fit to the superferromagnetism model (Equation 11). The order parameter vanishes at *T*_0_ ≈ 390 K, where the particles become superparamagnetic. For comparison, the Néel temperature of bulk hematite is about 955 K.

**Figure 7:**
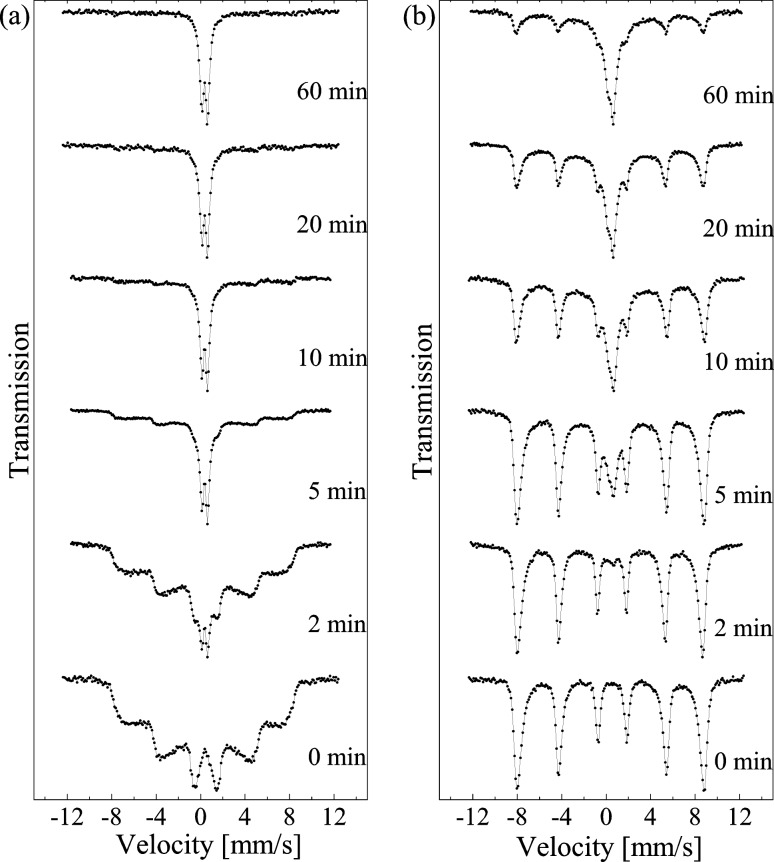
Mössbauer spectra of 8 nm hematite nanoparticles ground in a mortar with η-Al_2_O_3_ nanoparticles for the indicated periods of time. (a) Spectra obtained at room temperature. (b) Spectra obtained at 80 K. Reprinted with permission from Xu, M.; Bahl, C. R. H.; Frandsen, C.; Mørup, S. Inter-particle interactions in agglomerates of α-Fe_2_O_3_ nanoparticles: Influence of grinding, *J. Colloid Interface Science* **2004,*** 279* 132–136. Copyright (2004) by Elsevier.

The strength of interactions between nanoparticles is very sensitive to the method of sample preparation. For example, gentle grinding of nanoparticles in a mortar can have a dramatic influence on the relaxation behavior. This is illustrated in Figure 7, which shows Mössbauer spectra of samples of 8 nm hematite nanoparticles, prepared by drying aqueous suspensions of chemically prepared particles and after grinding for different periods of time together with nanoparticles of η-Al_2_O_3_ [45]. At room temperature, the spectrum of the as-prepared sample shows a sextet with very broad lines, typical for samples in which the superparamagnetic relaxation is suppressed by inter-particle interactions. At 80 K the spectrum consists of a sextet with relatively narrow lines. On grinding for only a few minutes the appearance of an intense doublet in the room-temperature spectra is observed. This indicates that the inter-particle interactions are strongly reduced. The spectra obtained at 80 K after grinding show a superposition of sextets and doublets typical for non-interacting or weakly interacting nanoparticles. After 60 min grinding, all particles are superparamagnetic at room temperature, and most of them also at 80 K. Thus, gentle grinding appears to separate strongly interacting nanoparticles. In later studies it has been shown that after strongly interacting nanoparticles have been dispersed by intense ultrasonic treatment, the magnetic interactions can be re-established by drying suspensions of the dispersed particles [46].

### Influence of inter-particle interactions on the spin structure in nanoparticles

The spin structure in nanoparticles may differ from that of the corresponding bulk materials, and magnetic inter-particle interactions can have a large influence on the spin orientation. In Mössbauer spectroscopy studies of magnetic materials, the spin orientation relative to the crystal axes may be studied by analyzing the quadrupole shift, ε of magnetically split spectra, which is given by the expression

[13]
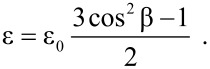


**Figure 8:**
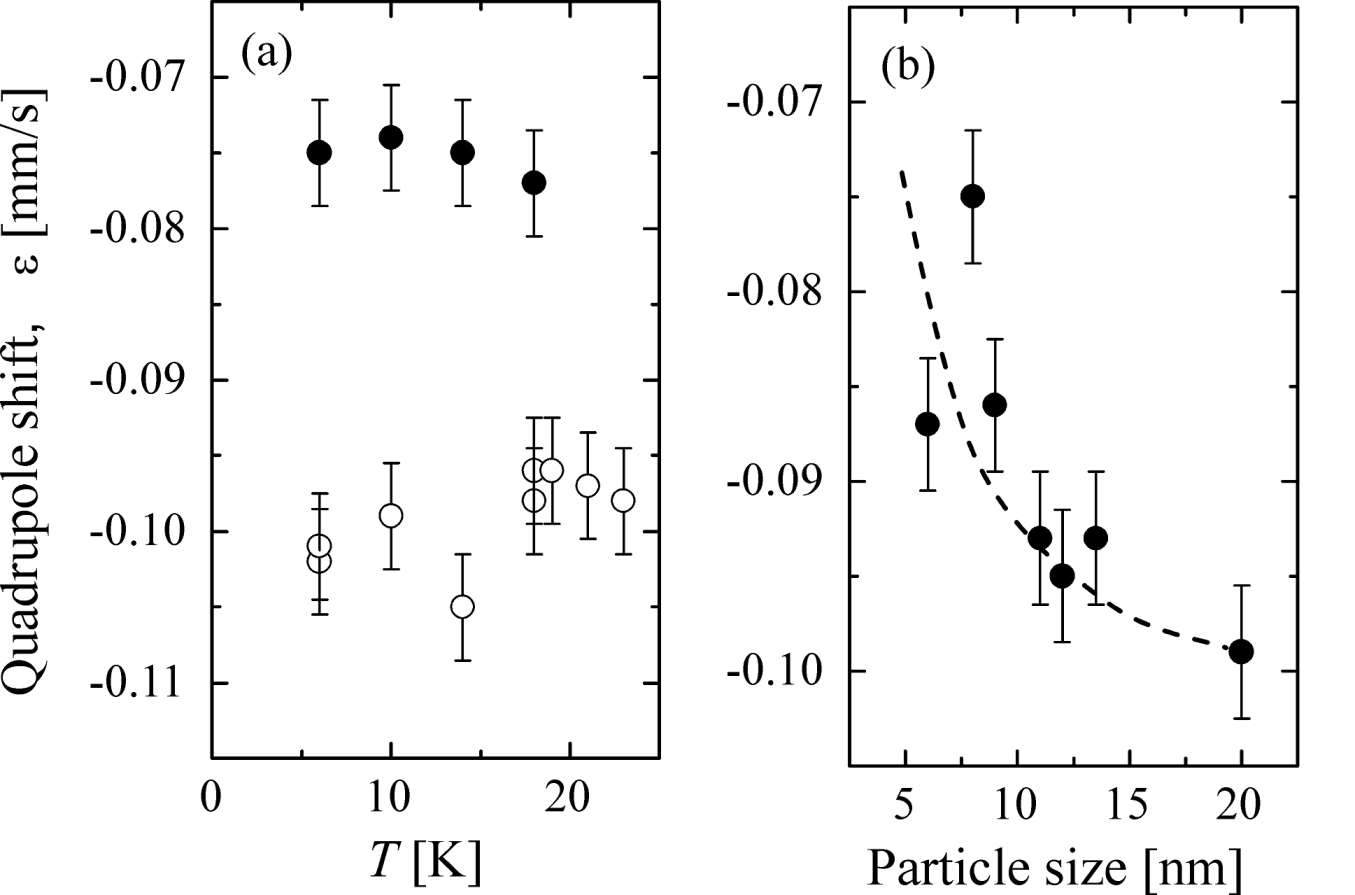
(a) The quadrupole shift of coated (open circles) and uncoated (solid circles) 8 nm hematite particles as a function of temperature. (b) The quadrupole shift of uncoated hematite nanoparticles at 20 K as a function of particle size. Reprinted from Frandsen, C.; Mørup, S. Spin rotation in α-Fe_2_O_3_ nanoparticles by interparticle interactions, *Phys. Rev. Lett*. **2005,** *94,* 027202. Copyright (2005) by the American Physical Society.

Here, β is the angle between the symmetry direction of the electric field gradient and the magnetic hyperfine field. In hematite, ε_0_ = 0.200 mm/s, and the symmetry direction of the electric field gradient is parallel to the [001] axis of the hexagonal unit cell. In non-interacting hematite nanoparticles and in bulk hematite above the Morin transition temperature (~263 K), the magnetic hyperfine field is perpendicular to this direction (β = 90°), resulting in a quadrupole shift of −0.100 mm/s. In samples of interacting hematite nanoparticles the absolute value of the quadrupole shift at low temperatures is slightly smaller (ε ≈ −0.075 mm/s in interacting 8 nm particles [36]). This is illustrated in Figure 8a and indicates a rotation of the spin direction, corresponding to β ≈ 75°, i.e., an out-of-plane spin rotation of about 15°, induced by inter-particle interactions.

The spin rotation can be explained by interactions between hematite nanoparticles for which the easy axis forms the angle θ_0_ with the interaction field. In this case Equation 9 should be replaced by

[14]
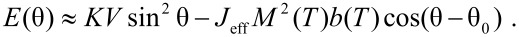


In the simple case when θ_0_ = 90° one can find the analytical solution for the value of θ, which gives the lowest energy:

[15]
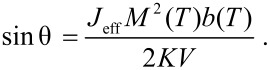


Figure 8b shows the quadrupole shift of uncoated hematite nanoparticles at 20 K as a function of particle size. There is an overall tendency that the deviation of ε from the bulk value decreases with increasing particle size, i.e., the rotation angle decreases with increasing particle size. This is at least qualitatively in agreement with the volume dependence of the rotation angle given by Equation 15.

In studies of interacting nanoparticles of hematite and NiO, a spin rotation much larger than 15° has been found. At low temperatures, the hematite particles showed quadrupole shifts up to around +0.16 mm/s, corresponding to β ≈ 21°, i.e., an out-of-plane spin rotation of about 69° [41]. Furthermore, the quadrupole shifts were found to decrease with increasing temperature. This is also in accordance with Equation 15, because of the decrease of the order parameter, *b*(*T*) with increasing temperature, as illustrated in Figure 6.

## Conclusion

During the first decades after the discovery of superparamagnetism, almost all experimental data for the magnetic dynamics of nanoparticles were analyzed by use of the theoretical models for non-interacting particles by Néel [7] and Brown [8]. However, in many more recent studies it has been realized that magnetic interactions between nanoparticles often play a crucial role. Long-range magnetic dipole interactions are important in samples of ferromagnetic and ferrimagnetic nanoparticles unless the particles are well separated. In samples with a high particle concentration, the inter-particle dipole interactions can result in formation of a collective state. If the particles are randomly distributed, the collective state can have many similarities to a spin glass. In other cases, for example, if the particles form chains, their magnetic moments may be aligned. Studies of antiferromagnetic particles have shown that exchange interactions between particles in close proximity can also result in the formation of a collective state at temperatures where the particles would be superparamagnetic if isolated. The temperature dependence of the order parameter is in accordance with a simple mean field theory. Studies of hematite nanoparticles have shown that exchange interactions between magnetic nanoparticles with different orientations of the easy axes can result in a rotation of the spin structure. Thus, systems of interacting magnetic nanoparticles show a rich variety of phenomena that are interesting both for fundamental scientific studies and for applications of magnetic nanoparticles in, e.g., magnetic data storage media.
